# Screening programmes and breast cancer mortality: an observational study of 194 countries

**DOI:** 10.2471/BLT.24.292529

**Published:** 2025-05-27

**Authors:** Syed Mahfuz Al Hasan, Debbie L Bennett, Adetunji T Toriola

**Affiliations:** a Division of Public Health Sciences, Department of Surgery, Washington University School of Medicine, 660 S Euclid Avenue, St Louis, MO 63110, United States of America (USA).; b Mallinckrodt Institute of Radiology, Washington University School of Medicine, St Louis, USA.

## Abstract

**Objective:**

To investigate the associations between national breast cancer screening programmes and breast cancer mortality globally.

**Methods:**

We collected data on breast cancer screening programmes and breast cancer mortality from the World Health Organization’s Global Health Observatory, the Global Burden of Diseases 2021 study and the Eurostat database. We assessed differences in breast cancer mortality between countries with regular and irregular screening programmes, adjusting for sociodemographic index. We calculated annual changes in breast cancer mortality from 2015 to 2021 and assessed differences in mortality changes between countries with regular and irregular screening programmes.

**Findings:**

Between 2015 and 2021, 94 of 194 countries reported having national breast cancer screening programmes. In 2021, countries with regular breast cancer screening programmes had 3.74 fewer deaths (95% uncertainty interval, UI: 1.69–5.81) per 100 000 population than countries with irregular screening programmes. This difference was more pronounced in women aged 50–74 years: 10.13 fewer deaths (95% UI: 4.47–15.80) per 100 000. From 2015 to 2021, the age-standardized mortality rate decreased by 1.02% (95% UI: 0.71–1.36) annually in countries with regular breast cancer screening programmes, whereas countries with irregular programmes had an annual increase of 0.45% (95% UI: 0.23–0.69). Higher breast cancer screening coverage rates were associated with lower mortality in the European region.

**Conclusion:**

Countries with breast cancer screening programmes had a significant reduction in breast cancer mortality. Reducing breast cancer mortality globally will require adopting national breast cancer screening programmes and increasing screening coverage, particularly among women aged 50–74 years.

## Résumé

**Objectif:**

Étudier les associations entre les programmes nationaux de dépistage du cancer du sein et la mortalité due à cette maladie à l’échelle mondiale.

**Méthodes:**

Nous avons recueilli des données sur les programmes de dépistage du cancer du sein et la mortalité due à cette maladie auprès de l’Observatoire mondial de la Santé de l’Organisation mondiale de la Santé, de l’étude Global Burden of Diseases 2021 et de la base de données d’Eurostat. Nous avons évalué les différences de mortalité due au cancer du sein entre les pays ayant mis en place des programmes de dépistage réguliers et ceux disposant de programmes irréguliers, en tenant compte de l’indice sociodémographique. Nous avons calculé les variations annuelles de la mortalité due au cancer du sein entre 2015 et 2021 et évalué les différences de variations de la mortalité entre les pays disposant de programmes de dépistage réguliers et irréguliers.

**Résultats:**

Entre 2015 et 2021, 94 pays sur 194 ont déclaré avoir mis en place des programmes nationaux de dépistage du cancer du sein. En 2021, ceux qui ont implémenté des programmes réguliers comptaient 3,74 décès de moins (intervalle d’incertitude à 95%: 1,69–5,81) pour 100 000 femmes que les pays ayant des programmes de dépistage irréguliers. Cette différence était plus prononcée chez les femmes de 50 à 74 ans: 10,13 décès en moins (intervalle d’incertitude à 95%: 4,47–15,80) par 100 000 femmes. Entre 2015 et 2021, le taux de mortalité standardisé selon l’âge a diminué chaque année de 1,02% (intervalle d’incertitude à 95%: 0,71–1,36) dans les pays disposant de programmes réguliers de dépistage du cancer du sein, tandis que ceux ayant mis en place des programmes irréguliers ont connu une augmentation annuelle de 0,45% (intervalle d’incertitude à 95%: 0,23–0,69). Des taux de couverture plus élevés pour le dépistage du cancer du sein étaient associés à une mortalité plus faible dans la région de l’Europe.

**Conclusion:**

Les pays disposant de programmes de dépistage du cancer du sein ont enregistré une réduction significative de la mortalité due au cancer du sein. Pour réduire ce taux à l’échelle mondiale, il faudra adopter des programmes nationaux de dépistage du cancer du sein et augmenter la couverture du dépistage, en particulier chez les femmes de 50 à 74 ans.

## Resumen

**Objetivo:**

Investigar la asociación entre los programas nacionales de cribado de cáncer de mama y la mortalidad por esta causa a nivel mundial.

**Métodos:**

Se recopilaron datos sobre programas de cribado de cáncer de mama y mortalidad por cáncer de mama del Observatorio Mundial de la Salud de la Organización Mundial de la Salud, del estudio *Global Burden of Diseases 2021* y de la base de datos de Eurostat. Se evaluaron las diferencias en la mortalidad por cáncer de mama entre países con programas de cribado regulares e irregulares, ajustando por el índice sociodemográfico. Se calcularon los cambios anuales en la mortalidad por cáncer de mama entre 2015 y 2021 y se analizaron las diferencias entre países con programas de cribado regulares e irregulares.

**Resultados:**

Entre 2015 y 2021, 94 de 194 países informaron tener programas nacionales de cribado de cáncer de mama. En 2021, los países con programas regulares de cribado de cáncer de mama registraron 3,74 muertes menos (intervalo de incertidumbre del 95%, UI: 1,69 a 5,81) por cada 100 000 habitantes en comparación con aquellos con programas irregulares. Esta diferencia fue más pronunciada en las mujeres de 50 a 74 años: 10,13 muertes menos (UI del 95%: 4,47 a 15,80) por cada 100 000. Entre 2015 y 2021, la tasa de mortalidad estandarizada por edad disminuyó un 1,02% anual (UI del 95%: 0,71 a 1,36) en los países con programas regulares de cribado, mientras que en los países con programas irregulares se observó un aumento anual del 0,45% (UI del 95%: 0,23 a 0,69). Se observó una asociación entre mayores tasas de cobertura del cribado y menor mortalidad en la Región Europea.

**Conclusión:**

Los países con programas de cribado de cáncer de mama presentaron una reducción significativa en la mortalidad por esta causa. La disminución global de la mortalidad por cáncer de mama requerirá la adopción de programas nacionales de cribado y el aumento de la cobertura, especialmente entre las mujeres de 50 a 74 años.

## ملخص

الغرض دراسة العلاقة بين البرامج الوطنية لفحص سرطان الثدي ومعدلات وفيات سرطان الثدي عالميًا.

الطريقة قمنا بجمع بيانات حول برامج فحص سرطان الثدي ومعدل الوفيات بسبب سرطان الثدي، من مرصد الصحة العالمي التابع لمنظمة الصحة العالمية، ودراسة العبء العالمي للأمراض لعام 2021، وقاعدة بيانات Eurostat. قمنا بتقييم الاختلافات في وفيات سرطان الثدي بين الدول التي تطبق برامج فحص منتظمة وغير منتظمة، مع مراعاة المؤشر الاجتماعي السكاني. قمنا باحتساب التغيرات السنوية في وفيات سرطان الثدي من عام 2015 إلى عام 2021، وتقييم الاختلافات في تغيرات الوفيات بين الدول التي تطبق برامج فحص منتظمة وغير منتظمة.

النتائج بين عامي 2015 و2021، أفادت 94 دولة من أصل 194 دولة بتطبيق برامج فحص وطنية لسرطان الثدي. في عام 2021، شهدت الدول التي لديها برامج منتظمة لفحص سرطان الثدي انخفاضًا في عدد الوفيات بمقدار 3.74 (فاصل عدم الثقة %95: 1.69 إلى 5.81) لكل 100000 نسمة مقارنة بالدول التي لديها برامج فحص غير منتظمة. وكان هذا الاختلاف أكثر وضوحًا لدى النساء اللواتي تتراوح أعمارهن بين 50 و74 عامًا: 10.13 حالات وفاة أقل (فاصل عدم الثقة %95: 4.47 إلى 15.80) لكل 100000 شخص. من عام 2015 إلى عام 2021، انخفض معدل الوفيات الموحد حسب العمر بنسبة %1.02 (فاصل عدم الثقة %95: 0.71 إلى 1.36) سنويًا في الدول التي لديها برامج منتظمة لفحص سرطان الثدي، بينما شهدت الدول التي لديها برامج غير منتظمة زيادة سنوية قدرها %0.45 (فاصل عدم الثقة %95: 0.23 إلى 0.69). ارتبطت معدلات تغطية فحص سرطان الثدي الأعلى بانخفاض معدل الوفيات في المنطقة الأوروبية.

الاستنتاج شهدت الدول التي لديها برامج فحص لسرطان الثدي انخفاضًا كبيرًا في وفيات سرطان الثدي. إن خفض معدل الوفيات الناجمة عن سرطان الثدي على مستوى العالم يتطلب اعتماد برامج وطنية لفحص سرطان الثدي وزيادة تغطية الفحص، وخاصة بين النساء في الفئة العمرية من 50 إلى 74 عاماً.

## 摘要

**目的:**

旨在探讨在全球范围内实施国家乳腺癌筛查计划与乳腺癌死亡率之间的关联性。

**方法:**

我们从世界卫生组织全球卫生观察站、2021 年全球疾病负担研究以及欧盟统计局数据库中收集了关于乳腺癌筛查计划和乳腺癌死亡率的数据。我们评估了实施定期筛查计划与实施非定期筛查计划的国家在乳腺癌死亡率方面的差异，并根据社会人口统计学指数进行了相应调整。我们计算了 2015 年至 2021 年期间在乳腺癌死亡率方面的年度变化，并评估了实施定期筛查计划与实施非定期筛查计划的国家在死亡率变化方面的差异。

**结果:**

在 2015 年至 2021 年期间，这 194 个国家中有 94 个报告称其实施了国家乳腺癌筛查计划。2021 年，与实施非定期筛查计划的国家相比，实施定期乳腺癌筛查计划的国家每 10 万人中的死亡人数减少了 3.74 例（95% 不确定性区间 (UI)：1.69-5.81）。在年龄介于 50 至 74 岁之间的女性中，这一差异更加明显：每 10 万人中的死亡人数减少了 10.13 例（95% UI：4.47-15.80）。在 2015 年至 2021 年期间，实施定期乳腺癌筛查计划的国家的年龄标化死亡率每年下降 1.02%（95% UI：0.71-1.36），而实施非定期筛查计划的国家的年龄标化死亡率则每年增长 0.45%（95% UI：0.23-0.69）。在欧洲区域，乳腺癌筛查覆盖率越高，则死亡率越低。

**结论:**

在实施乳腺癌筛查计划的国家中，乳腺癌死亡率显著降低。若要降低全球范围内的乳腺癌死亡率，各国必须落实国家乳腺癌筛查计划并扩大筛查覆盖范围，特别是对于年龄介于 50 至 74 岁之间的女性。

## Резюме

**Цель:**

Изучить связь между наличием национальных программ скрининга на рак молочной железы и показателями смертности от данного заболевания в глобальном масштабе.

**Методы:**

Авторы собрали данные о программах скрининга на рак молочной железы и о показателях смертности от данного заболевания по данным Глобальной обсерватории здравоохранения Всемирной организации здравоохранения, исследования глобального бремени заболеваний за 2021 г. и базы данных Eurostat. Были изучены различия в смертности от рака молочной железы в странах с программами регулярного и нерегулярного скрининга с поправкой на социально-демографические показатели. Авторы рассчитали ежегодные изменения в смертности от рака молочной железы в период между 2015 и 2021 годами и оценили различия в динамике смертности для стран с программами регулярного и нерегулярного скрининга.

**Результаты:**

В период с 2015 по 2021 год 94 из 194 стран сообщили о наличии у них национальных программ скрининга на рак молочной железы. В 2021 году страны с программами регулярного скрининга на рак молочной железы имели на 3,74 случая смерти меньше (95%-й интервал неопределенности, ИН: 1,69–5,81) на 100 000 населения, чем страны с программами нерегулярного скрининга. Эта разница была сильнее выражена для женщин в возрасте 50–74 лет: смертность меньше на 10,13 случая (95%-й ИН: 4,47–15,80) на 100 000 населения. В период с 2015 по 2021 год стандартизированная по возрасту смертность сократилась на 1,02% (95%-й ИН: 0,71–1,36) в год в странах с программами регулярного скрининга на рак молочной железы, тогда как для стран с программами нерегулярного скрининга наблюдался среднегодовой рост в 0,45% (95%-й ИН: 0,23–0,69). В Европейском регионе более высокая степень охвата скринингом ассоциировалась с меньшей смертностью от рака молочной железы.

**Вывод:**

Для стран, в которых имеются программы скрининга на рак молочной железы, характерно значительное снижение смертности от данного заболевания. Снижение показателей смертности от рака молочной железы во всем мире потребует введения национальных программ скрининга и расширения охвата, в частности среди женщин в возрасте 50–74 лет.

## Introduction

Breast cancer is a great global public health challenge as it is the most prevalent cancer in women in most countries, and the leading or second leading cause of female cancer-related deaths in 94.54% (173/183) of countries.^1^ In 2020, 2.26 million women were diagnosed with breast cancer, with 0.68 million deaths globally.^2^ The burden of breast cancer is predicted to increase to more than 3 million new cases and 1 million deaths every year by 2040.^2^

In response to the need for proactive intervention, several countries have implemented breast cancer screening programmes.^3^ In 2021, 63.40% (123) of the 194 World Health Organization (WHO) Member States reported having an existing national breast cancer screening programme.^4^ Effective screening programmes lead to early detection and diagnosis, which play crucial roles in reducing breast cancer mortality.^5^^–^^13^ While previous research has examined the association between breast cancer screening and breast cancer mortality, research gaps exist on the association between national breast cancer screening programmes and mortality at the global level. A global analysis will provide a comprehensive understanding of the effect of screening programmes on breast cancer mortality in countries with widely varying sociodemographic and economic make-up. This knowledge is crucial for policy-makers and health workers, allows assessment of the effectiveness of current programmes and identifies areas that need improvement.

We aimed to evaluate the associations between national breast cancer screening programmes and breast cancer mortality globally.

## Methods

### Data sources

We compiled our data set from three global databases: the Global Burden of Diseases, Injuries and Risk Factors Study (GBD) 2021 database;^14^ the WHO Global Health Observatory database;^15^ and the Eurostat Database.^16^ Our research complies with the Guidelines for Accurate and Transparent Health Estimates Reporting (GATHER)^17^ recommendations (completed GATHER checklist available in the online data repository).^18^

#### GBD database

We obtained annual age-standardized and age-specific breast cancer mortality data in females for 1990–2021, at regional and country levels, from the GBD 2021 study database. This study assesses the global burden of diseases, injuries and risk factors by age, sex and location. In 2021, the GBD study covered 371 diseases and injuries and 88 risk factors, and spanned seven super-regions, 21 regions, 204 countries and territories and 811 subnational locations, accumulating data from 1990 onwards.^19^

#### WHO Global Health Observatory

Information on breast cancer screening programmes for 2015, 2017, 2019 and 2021 for 194 countries is available in the WHO Global Health Observatory database. This database includes various indicators of health in several countries, including cause-specific mortality and health-service availability.^15^ Countries are categorized as having a national screening programme if they responded yes to whether they had a screening programme for breast cancer targeting the general population (mammographic screening or clinical breast examination). The programme could be offered either through organized population-based screening or opportunistic screening.^4^

#### Eurostat database

We also studied the association between national mammographic screening and breast cancer mortality. Because of limited data on mammographic screening coverage across all countries, we limited this analysis to the European region, where data were available from 2013 to 2022 within the Eurostat database. This database provides reliable and high-quality data on social, economic and environmental developments within the European region.^16^

### Compilation of data sets

Information on breast cancer screening programmes was available for 194 countries from 2015 to 2021. Breast cancer mortality data (all-age deaths, age-standardized and age-specific mortality rates, and mortality-to-incidence ratios) were available for 204 countries from 1990 to 2021. We therefore evaluated the associations between breast cancer screening programmes and breast cancer mortality from 2015 to 2021. We merged the breast cancer screening programmes and breast cancer mortality databases, using the country name as the main variable for merging. We had 194 countries for this analysis. We compiled a separate data set for the European region based on information on mammographic screening coverage for 31 European countries from 2013 to 2022 (available in online repository).^18^ We excluded North Macedonia and Romania because they had low coverage rates (< 1.0%). We averaged the mammographic screening coverage data for 2013–2022. We then stratified the European countries into tertiles based on their mammographic screening coverage distributions. We compared breast cancer mortality rates across these tertiles from 2013 to 2021.

### Country grouping

We categorized countries into three groups based on the presence of breast cancer screening programmes. Group 1 (reference group) were countries with regular breast cancer screening programmes (consistent population-based screening programmes in 2015, 2017, 2019 and 2021). Group 2 were countries with no screening programmes (consistently reported no population-based screening programmes in 2015, 2017, 2019 and 2021). Group 3 were countries with random screening programmes (inconsistent population-based screening programmes in 2015, 2017, 2019 and 2021). We combined groups 2 and 3 and classified them as countries with irregular screening programmes.

### Mortality estimates in GBD

The GBD estimation framework and its metric calculations are described in the GBD 2021 publications.^20^^,^^21^ Briefly, the GBD cancer mortality estimation process has two main steps. First, estimation of cancer mortality-to-incidence ratios from cancer registries is needed. By calculating these ratios, existing incidence data can be used to estimate mortality rates where mortality data are limited. The mortality-to-incidence ratios are then modelled using a space–time Gaussian process regression approach, considering matched incidence and mortality data from cancer registries and the GBD-estimated health-care access and quality index as covariates.^22^ These estimated ratios are used to convert cancer registry incidence data for mortality modelling. The second step is estimating cancer mortality using the cause-of-death ensemble model. In this model, data are combined from vital registration systems, cancer registries and verbal autopsy reports to estimate mortality in various submodels. Model construction and performance are assessed through predictive validity tests. Sex-specific cause-of-death ensemble models are used to generate mortality estimates for specific locations, years and age groups.^20^^,^^21^ These estimates are then adjusted to align with total mortality estimates for all causes of death separately calculated in GBD 2021.

### Sociodemographic index

We adjusted for breast cancer mortality rates using the sociodemographic index to account for country-level development and minimize confounding due to socioeconomic factors.^23^ The sociodemographic index is a composite indicator of lag-distributed income per capita, average educational attainment for individuals older than 15 years and total fertility rate among individuals younger than 25 years. To ensure comparability across countries, each of the values of these indicators was rescaled from 0 (representing the lowest observed value) to 1 (representing the highest observed value), on the basis of country-specific data to allow for standardization.^23^ The final value of the country-level sociodemographic index was calculated by taking the geometric mean of these rescaled indices.

### Statistical analyses

We used a bootstrap independent sample *t*-test to compare age-standardized breast cancer mortality rates between countries with regular and irregular screening programmes. We determined statistical significance by bootstrapping on about 1000 random samples. We calculated the 95% uncertainty interval (UI) of the estimates based on a bias-corrected and accelerated procedure using bootstrap samples. In addition to the *t*-test, we used a general linear model, adjusting for sociodemographic differences across the countries, to compare breast cancer mortality between countries with regular and irregular screening programmes. We corrected for multiple tests using the Bonferroni adjustment to control the familywise error rate.

For the analysis of annualized per cent changes in breast cancer mortality for age (age-specific and age-standardized mortality), we used joinpoint regression analysis (Joinpoint regression programme, version 4.9.0.0, National Institutes of Health, Bethesda, United States of America).^24^ We began with a null model assuming no joinpoints. We tested this model against alternative models that allowed for the possibility of one or more joinpoints (maximum of five). We assessed the statistical significance of the alternative model; if the null model was rejected indicating a better fit, it was replaced by the alternative model.^24^ The data-driven Bayesian information criteria method was used for optimal model selection. Specifically, the optimal model was the model with the minimum weighted value of Bayesian information criteria.^25^ Using the optimal model, we estimated the annual per cent change in breast cancer mortality for each of the seven super-regions, 21 regions and 194 countries. Additionally, we analysed the age-specific trends in breast cancer mortality for each of the 194 countries. We calculated the average annual per cent change in breast cancer mortality as a geometric weighted average of the estimated annual per cent changes across various segments of the underlying trends.^26^ We estimated the average annual per cent change for 2015–2021, the years when data on breast cancer screening programmes were available.

As we identified sudden changes in breast cancer mortality during the coronavirus disease 2019 (COVID-19) pandemic (2020–2021) in 21 countries that could skew the annualized per cent changes in mortality rates from 2015 to 2021, we conducted a sensitivity analysis to address this concern by excluding outlier countries (available in online repository).^18^ We performed other analyses on the outlier countries to assess the difference in breast cancer mortality between countries with regular and irregular screening programmes (available in online repository).^18^ Our sensitivity analysis did not show any significant variations, confirming the robustness of our overall findings.

To provide a clearer pre-pandemic baseline, we calculated an additional set of annualized per cent changes in mortality from 2015 to 2019 in the 194 countries. We undertook a comparative analysis of breast cancer mortality between countries with regular and irregular breast cancer screening programmes for both periods: 2015–2021 and 2015–2019. This dual-period analysis allowed us to discern the potential effect of screening programmes on breast cancer mortality trends, independent of disruptions caused by the COVID-19 pandemic.

## Results

**Fig. 1:**
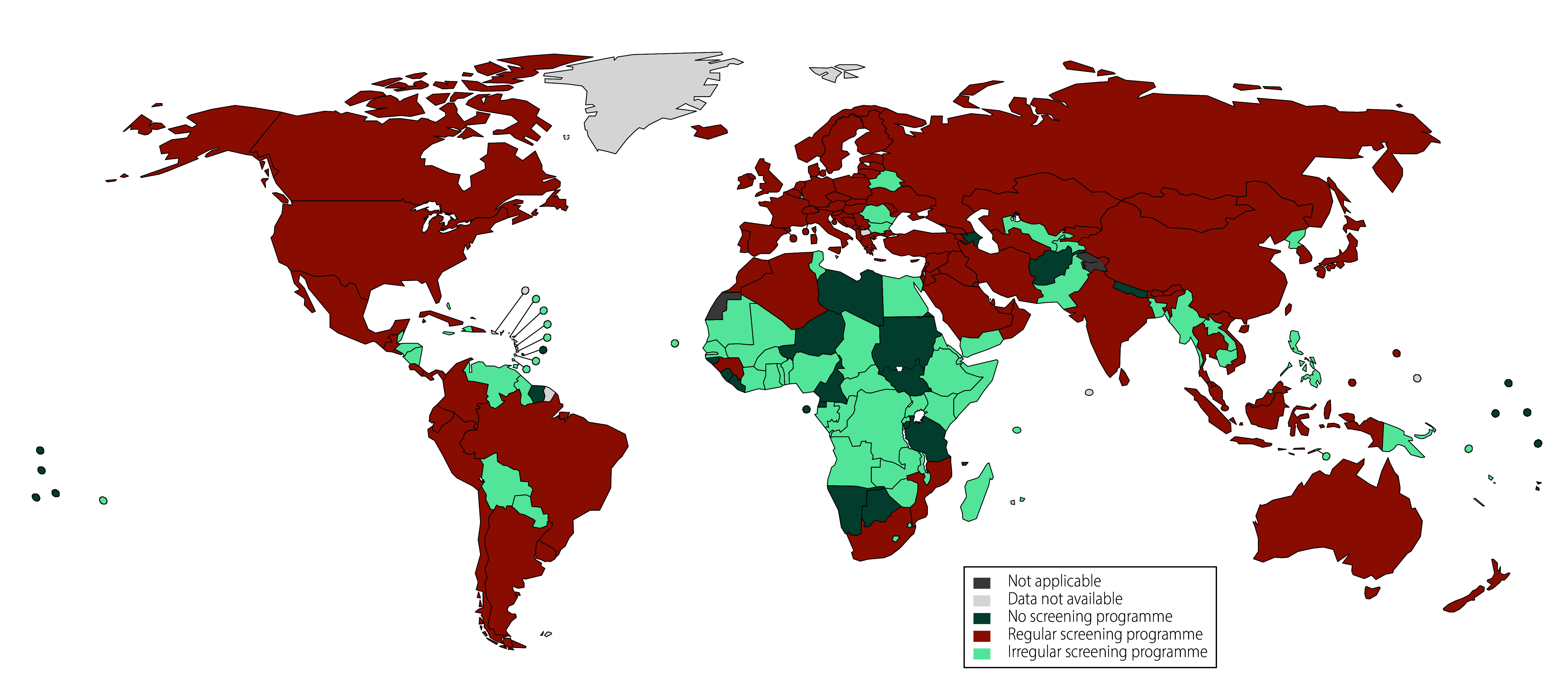
Distribution of countries with national breast cancer screening programmes, 2015–2021

Note: countries with responses of ‘no data received,’ ‘do not know,’ or ‘no response’ in the WHO Global Health Observatory database for any year between 2015 and 2021 were classified as having an irregular screening programme.

Source: World Health Organization Global Health Observatory Database.^15^

**Fig. 2:**
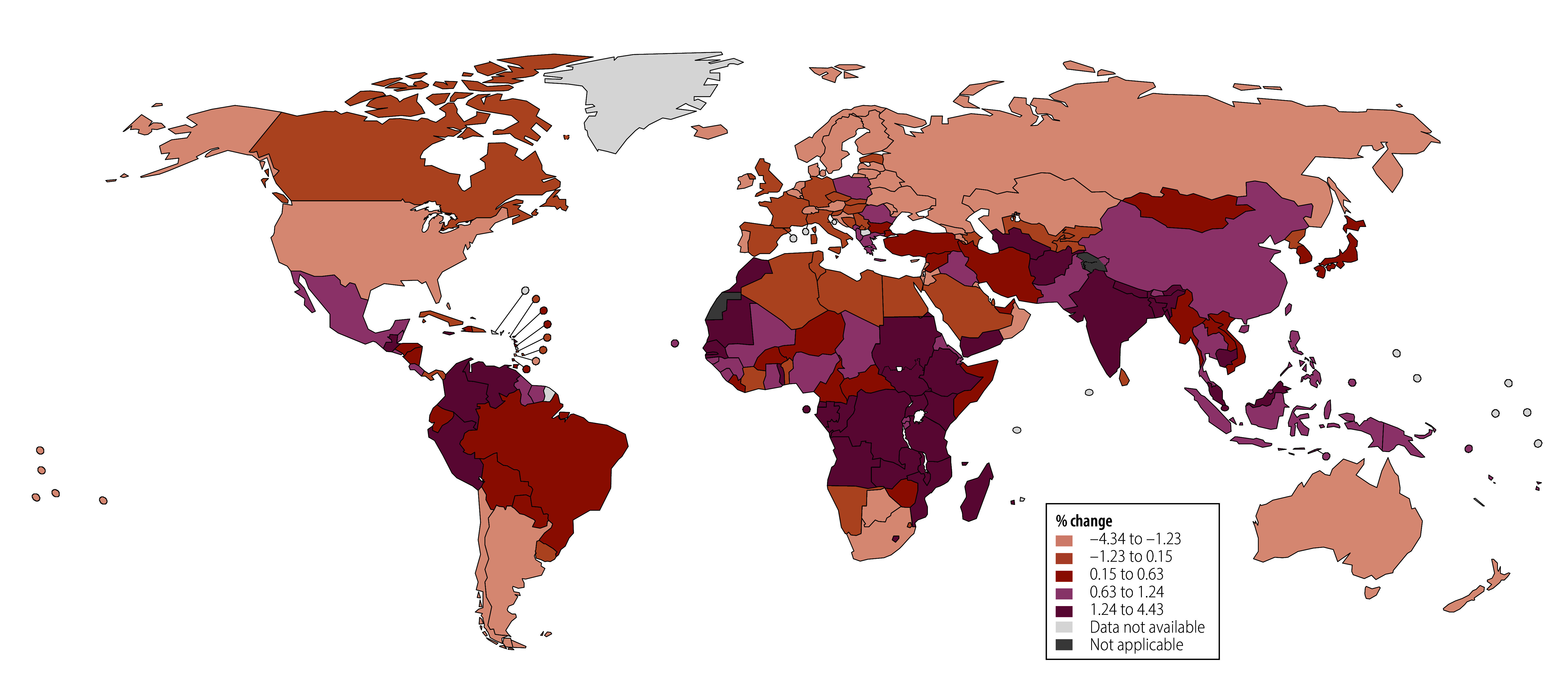
Distribution of countries by quintile of annualized per cent changes in breast cancer mortality rate, 2015–2019

**Fig. 3:**
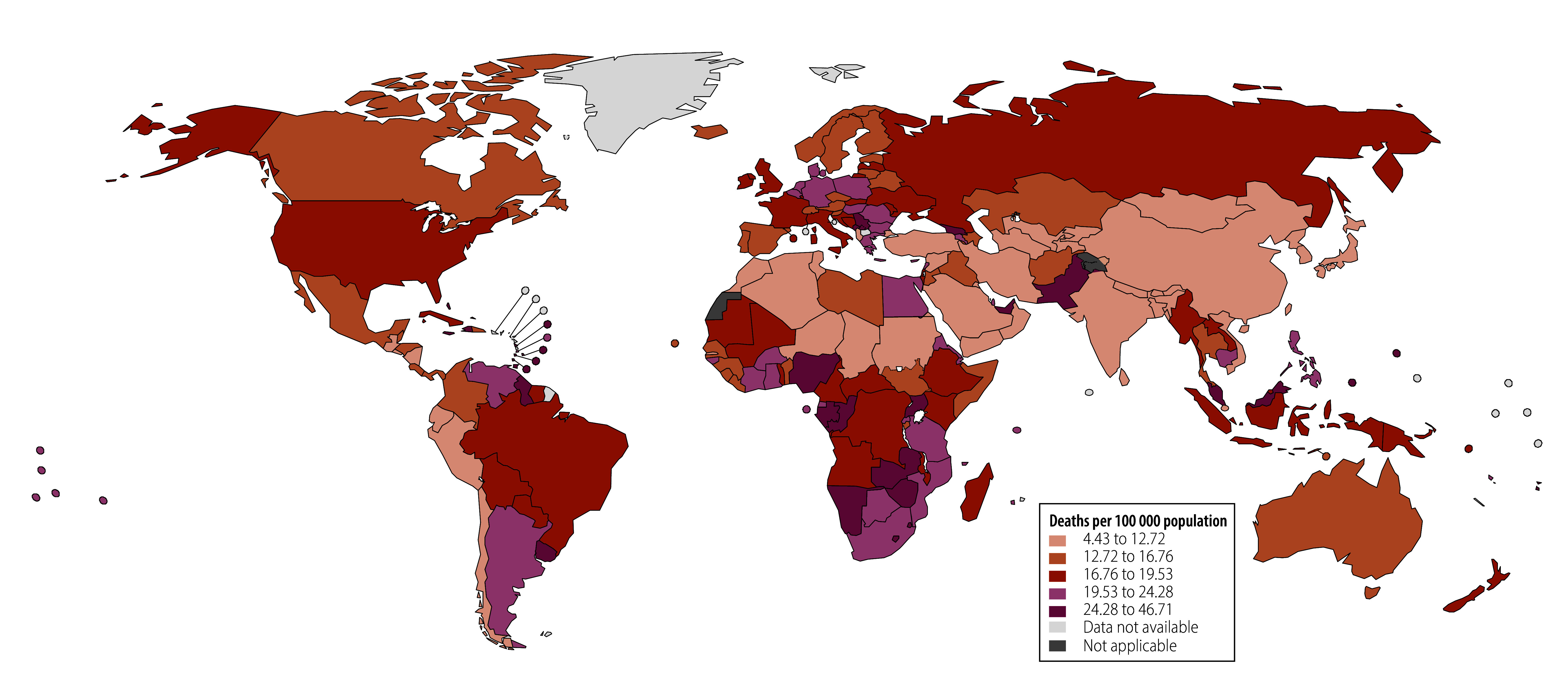
Distribution of countries by quintile of mean age-standardized breast cancer mortality rate during the COVID-19 pandemic, 2020–2021

Between 2015 and 2021, 94 of 194 countries had a population-based breast cancer screening programme (Fig. 1 and online repository).^18^ Overall, 39.69% (77/194) of countries reported inconsistently on the presence of a population-based breast cancer screening programme (online repository),^18^ whereas 11.86% (23/194) had no population-based breast cancer screening programme (online repository).^18^ The distribution of countries by quintile of annualized per cent changes in breast cancer mortality from 2015 to 2019 and average mortality during the COVID-19 pandemic is presented in Fig. 2 and Fig. 3, respectively. 

COVID-19: coronavirus disease 2019.

**Fig. 4:**
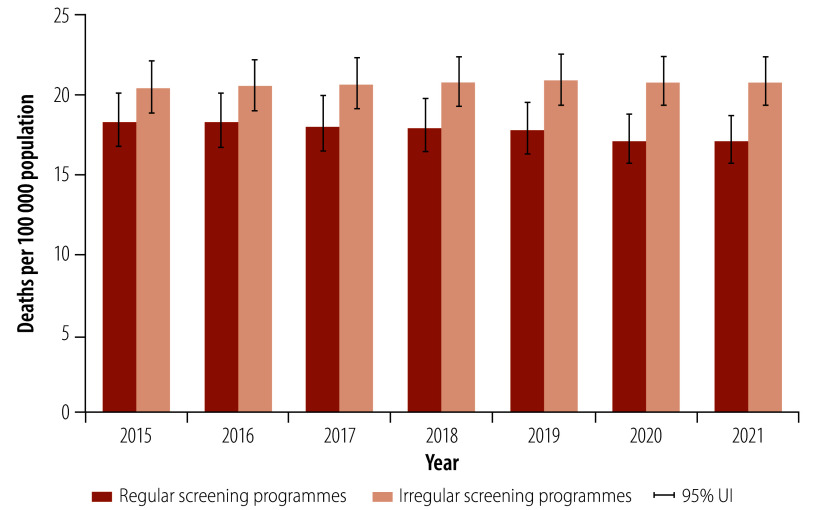
Mean age-standardized breast cancer mortality rates in countries with regular and irregular national breast cancer screening programmes, by year, 2015–2021

**Fig. 5:**
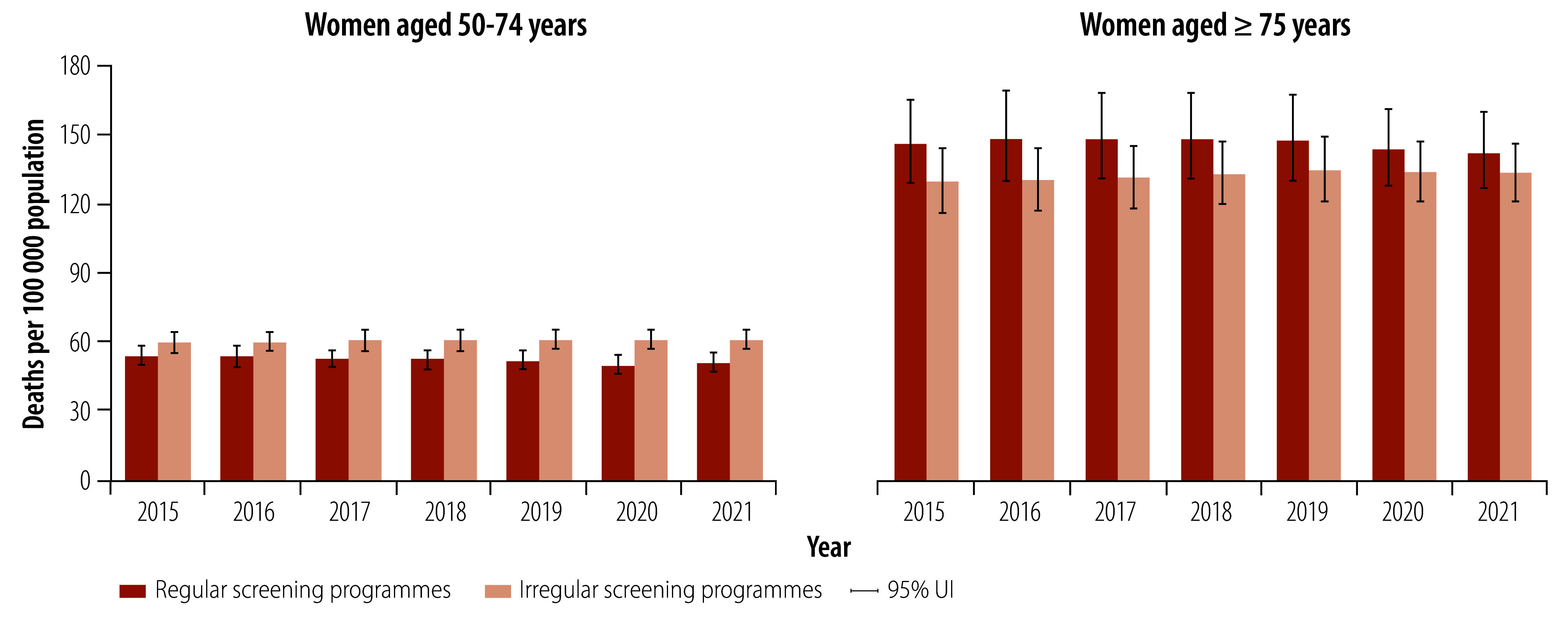
Age-specific breast cancer mortality rates in countries with regular and irregular national breast cancer screening programmes, by age group and year

**Fig. 6:**
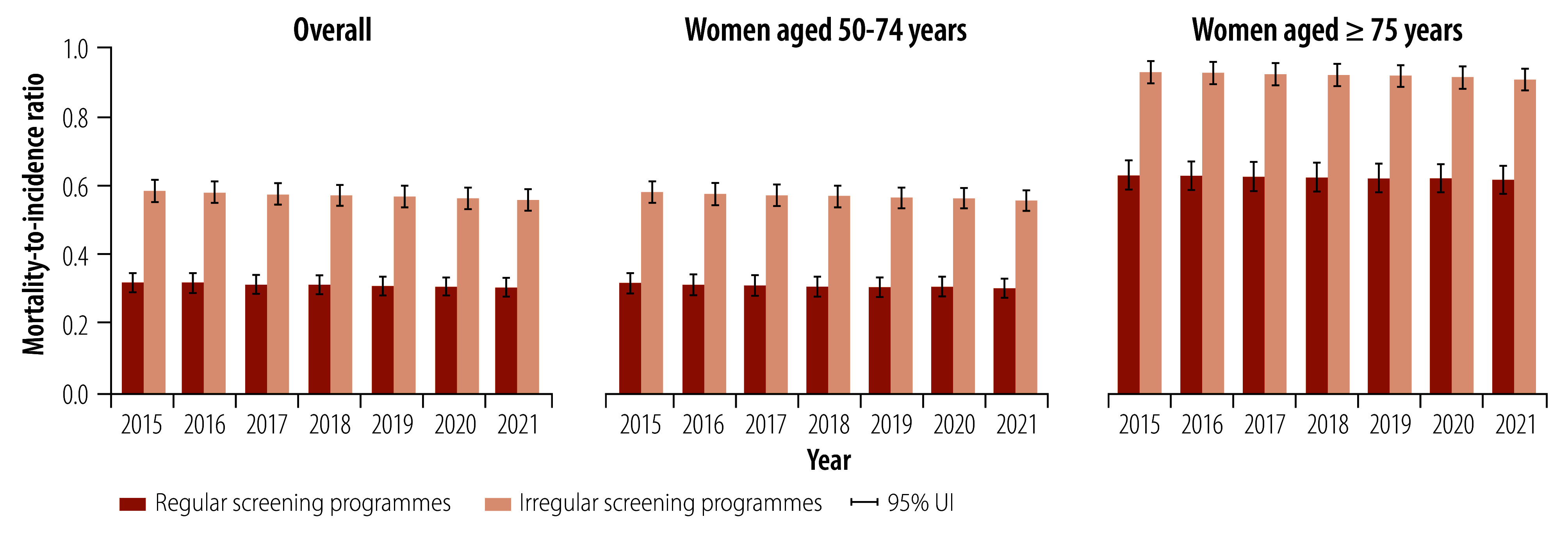
Mortality-to-incidence ratio in countries with regular and irregular national breast cancer screening programmes, overall and by age group, by year

Countries with regular breast cancer screening programmes had lower age-standardized mortality rates than countries with irregular screening programmes (Fig. 4). In 2021, countries with regular screening programmes had about 3.74 (95% UI: 1.69–5.81) fewer deaths per 100 000 population than countries with irregular breast cancer screening programmes: 17.07 deaths (95% UI: 15.74–18.67) per 100 000 versus 20.81 deaths (95% UI: 19.56–22.09) per 100 000 (Fig. 4). The reduction in mortality rates was particularly pronounced in women aged 50–74 years. For example, in 2021, countries with regular breast cancer screening programmes had 10.13 fewer deaths (95% UI: 4.47–15.80) per 100 000 population than countries without regular breast cancer screening programmes: 50.52 deaths (95% UI: 46.93–54.39) per 100 000 versus 60.65 deaths (95% UI: 56.89–64.38) per 100 000 (Fig. 5). Moreover, countries with regular screening programmes had significantly lower mortality-to-incidence ratios than countries with irregular screening programmes in both women aged 50–74 years (0.30; 95% UI: 0.28–0.33 versus 0.56; 95% UI: 0.53–0.58) and women aged 75 years or older (0.61; 95% UI: 0.57–0.65 versus 0.90; 95% UI: 0.87–0.93) in 2021 (Fig. 6).

UI: uncertainty interval.

Notes: the difference in mortality between countries with regular and irregular national breast cancer screening programmes was statistically significant in all years except 2015 (*P* < 0.05).

UI: uncertainty interval.

Notes: the difference in mortality between countries with regular and irregular national breast cancer screening programmes in women aged 50–74 years was statistically significant in 2017–2021 (*P* < 0.05).

UI: uncertainty interval.

Notes: the difference in ratios between countries with regular and irregular national breast cancer screening programmes was statistically significant in both age groups and all years (*P* < 0.01).

**Fig. 7:**
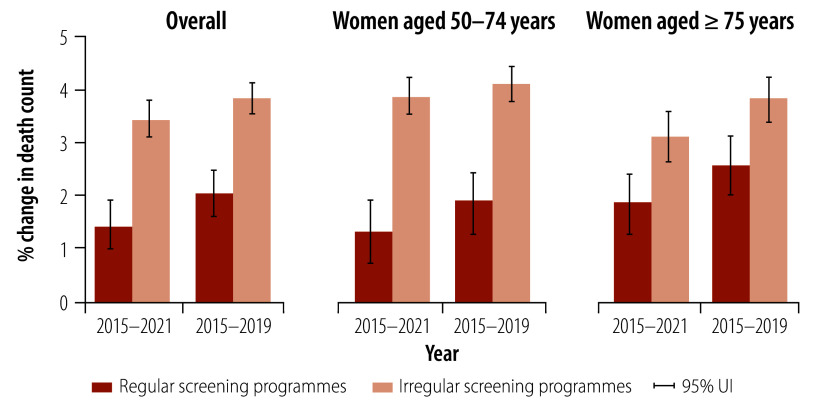
Annualized per cent changes in breast cancer deaths between countries with regular and irregular national breast cancer screening programmes, overall and by age group, 2015–2021 and 2015–2019

**Fig. 8:**
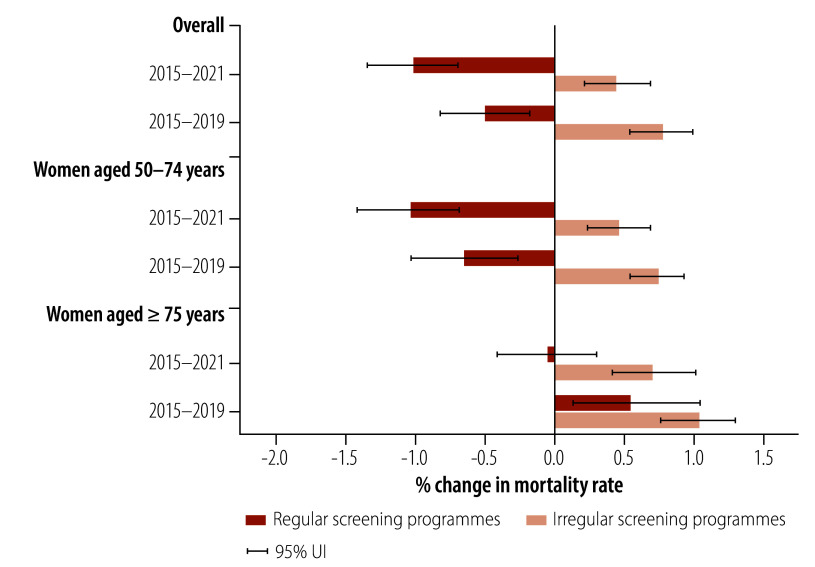
Annualized per cent changes in breast cancer mortality rates between countries with regular and irregular national breast cancer screening programmes, overall and by age group, 2015–2021 and 2015–2019

Notes: the estimates were calculated from 1000 bootstrap samples. The 95% UI error bar was calculated based on a bias-corrected and accelerated procedure.

Countries with regular screening programmes had a lower annual increase in breast cancer deaths of 1.45% (95% UI: 1.01–1.92) during 2015–2021. In contrast, countries with irregular screening programmes had a comparatively higher annual increase in breast cancer deaths of 3.43% (95% UI: 3.80–3.11; Fig. 7). Furthermore, the age-standardized mortality rate fell by 1.02% (95% UI: 0.71–1.36) annually during 2015–2021 in countries with regular breast cancer screening programmes, whereas countries with irregular screening programmes experienced a 0.45% (95% UI: 0.23–0.69) annual increase (Fig. 8). The difference in the annual changes in breast cancer mortality rates between countries with regular and irregular screening programmes was narrower before the COVID-19 pandemic (2015–2019): reduced by 0.50% (95% UI: 0.18–0.82) versus increased by 0.78% (95% UI: 0.55–0.99; Fig. 8). Annualized changes in age-standardized breast cancer mortality rates by country are presented in the online repository.^18^

UI: uncertainty interval.

Notes: the estimates were calculated from 1000 bootstrap samples. The 95% UI error bar was calculated based on a bias-corrected and accelerated procedure.

Table 1 shows regional variations in breast cancer screening programmes and breast cancer mortality rates. In 2021, compared with countries with irregular screening programmes, countries with regular screening programmes had a reduction in breast cancer mortality; however, the observed differences across all GBD super-regions were not significant except for Latin America and the Caribbean, and South-East Asia, East Asia, and Oceania super regions (Table 1). In Latin America and the Caribbean, countries with regular breast cancer screening programmes had 10.00 fewer deaths (95% UI: 5.29–14.71) per 100 000 than countries with irregular screening programmes. Similarly, in South-East Asia, East Asia and Oceania, 6.14 fewer deaths (95% UI: 0.88–11.39) per 100 000 occurred in countries with regular screening programmes than in countries with irregular screening programmes. In 2021, these regional reductions in breast cancer mortality in countries with regular screening programmes were more evident in women aged 50–74 years; for example, Latin America and the Caribbean had 29.48 fewer deaths (95% UI: 16.83–42.12) per 100 000; and South-East Asia, East Asia and Oceania regions had 17.18 fewer deaths (95% UI: 3.08–31.28) per 100 000 than countries with irregular screening programmes (Table 1).

**Table 1:** Breast cancer mortality across GBD super-regions, by regularity of breast cancer screening programme, 2021

Super-region	Mortality rate per 100 000 population (95% UI)^a^			*P^b^*
	Countries with regular screening programmes	Countries with irregular screening programmes	Difference in mortality rates	
**Age-standardized mortality rate**				
Central Europe, Eastern Europe and Central Asia	14.14 (11.76 to 16.88)	13.90 (11.02 to 17.28)	0.24 (−5.49 to 5.97)	0.935
High-income region	11.31 (8.10 to 14.25)	11.72 (7.37 to 14.93)	−0.41 (−9.47 to 8.64)	0.929
Latin America and the Caribbean	13.69 (12.41 to 14.92)	23.69 (20.91 to 26.64)	−10.00 (−14.71 to −5.29)	< 0.001
North Africa and the Middle East	12.74 (8.72 to 17.08)	14.63 (10.43 to 19.75)	−1.90 (−8.20 to 4.41)	0.553
South Asia	13.93 (12.25 to 15.32)	20.12 (11.00 to 34.20)	−6.19 (−17.55 to 5.17)	0.283
South-East Asia, East Asia and Oceania	20.26 (11.66 to 30.22)	26.40 (22.38 to 30.42)	−6.14 (−11.39 to −0.88)	0.022
Sub-Saharan Africa	26.00 (22.90 to 30.40)	26.28 (23.77 to 28.93)	−0.28 (−7.70 to 7.15)	0.942
**Age-specific mortality rate, women aged 50–74 years**				
Central Europe, Eastern Europe, and Central Asia	49.32 (41.43 to 57.14)	47.90 (38.49 to 59.24)	1.42 (−13.95 to 16.80)	0.855
High-income region	34.88 (27.17 to 42.61)	36.72 (22.91 to 48.17)	−1.83 (−26.13 to 22.46)	0.882
Latin America and the Caribbean	40.71 (36.85 to 44.67)	70.19 (61.94 to 78.11)	−29.48 (−42.12 to −16.83)	< 0.001
North Africa and the Middle East	31.65 (22.42 to 40.22)	41.66 (30.67 to 56.22)	−10.01 (−26.92 to 6.90)	0.244
South Asia	40.97 (35.97 to 45.16)	58.50 (33.21 to 99.96)	−17.52 (−48.01 to 12.96)	0.258
South-East Asia, East Asia, and Oceania	57.03 (36.85 to 76.51)	74.21 (64.21 to 83.58)	−17.18 (−31.28 to −3.08)	0.017
Sub-Saharan Africa	71.84 (63.47 to 82.84)	74.56 (67.92 to 82.14)	−2.72 (−22.65 to 17.22)	0.788
**Age-specific mortality rate, women aged ≥ 75 years**				
Central Europe, Eastern Europe and Central Asia	98.42 (74.42 to 124.08)	70.54 (38.70 to 110.02)	27.88 (−28.80 to 84.55)	0.333
High-income region	109.88 (78.51 to 140.88)	76.68 (11.90 to 144.85)	33.20 (−56.38 to 122.77)	0.466
Latin America and the Caribbean	93.28 (78.74 to 111.11)	165.42 (141.94 to 187.35)	−72.14 (−118.75 to −25.53)	0.003
North Africa and the Middle East	94.55 (49.84 to 154.14)	95.05 (58.68 to 140.00)	−0.50 (−62.85 to 61.85)	0.987
South Asia	92.11 (76.19 to 116.79)	125.74 (44.87 to 217.05)	−33.64 (−146.02 to 78.74)	0.555
South-East Asia, East Asia and Oceania	127.40 (58.84 to 209.73)	170.90 (134.80 to 208.90)	−43.50 (−95.48 to 8.48)	0.100
Sub-Saharan Africa	207.54 (171.72 to 262.34)	195.21 (173.53 to 223.01)	12.32 (−61.15 to 85.79)	0.741

GBD: Global Burden of Disease Study; UI: uncertainty interval.

^a^ Estimates were adjusted for differences in sociodemographic index across the countries.

^b^ *P* ≤ 0.05 was statically significant. For multiple comparisons, we used Bonferroni adjustments.

**Fig. 9:**
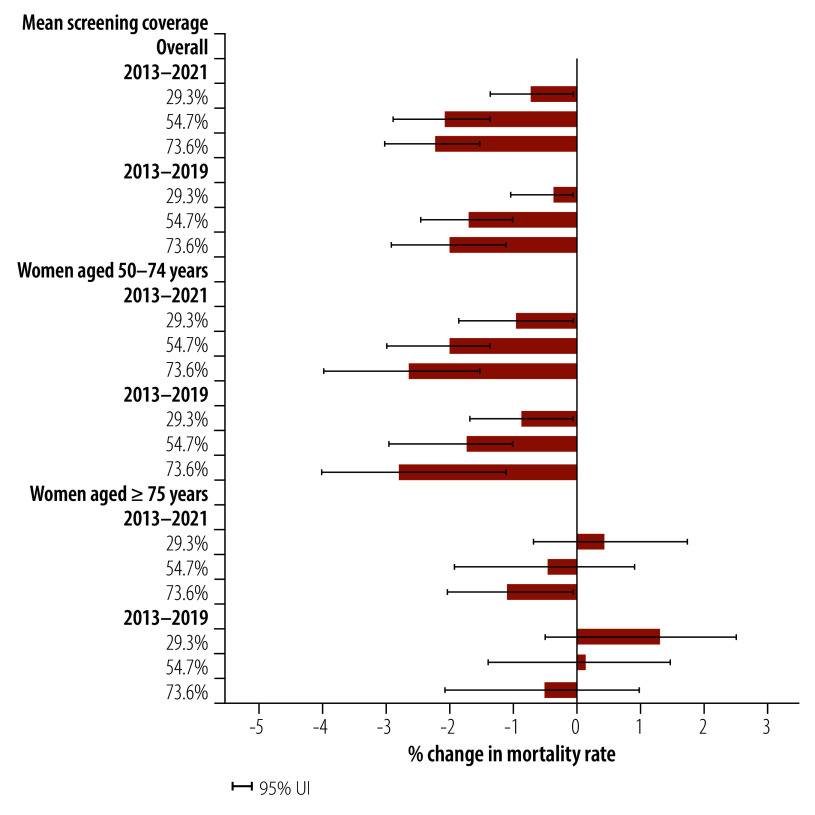
Annualized per cent changes in breast cancer mortality rates in European Region, overall and by age group, by mean mammogram screening coverage, 2013–2021 and 2013–2019

Note: the estimates were calculated from 1000 bootstrap samples. The 95% UI error bar was calculated based on a bias-corrected and accelerated procedure. Countries included are Austria, Belgium, Bulgaria, Croatia, Cyprus, Czechia, Denmark, Estonia, Finland, France, Germany, Hungary, Iceland, Ireland, Italy, Latvia, Liechtenstein, Lithuania, Luxembourg, Malta, Montenegro, Kingdom of the Netherlands, Norway, Poland, Serbia, Slovakia, Slovenia, Sweden and Türkiye.

Mammographic screening coverage and breast cancer mortality rates in the European region are presented in Fig. 9. Countries with higher breast cancer screening rates had a greater reduction in age-standardized breast cancer mortality than countries with lower screening rates. Specifically, countries with a mean screening coverage rate of 73.6% had an annual reduction of 2.24% (95% UI: 1.53–3.03) in breast cancer mortality from 2013 to 2021, whereas countries with 29.3% screening coverage had a reduction rate of 0.74% (95% UI: 0.09–1.35). The effect was particularly prominent in women aged 50–74 years, in whom the age-specific mortality rate fell by 2.65% (95% UI: 1.56–3.98) annually in countries with a mean screening coverage rate of 73.6% compared with a 0.98% (95% UI: 0.10–1.86) annual reduction in countries with a rate of 29.3%.

## Discussion

We show that breast cancer mortality was lower in countries with regular breast cancer screening programmes than in countries with irregular programmes. Countries with regular breast cancer screening programmes also experienced an annual reduction in the age-standardized mortality rate compared with countries with irregular programmes. This reduction was prominent in women aged 50–74 years. These findings highlight the importance of implementing and maintaining national breast cancer screening programmes to reduce breast cancer mortality.

The main objective of breast cancer screening is to decrease mortality and minimize the negative impact of advanced disease by identifying breast cancer early in asymptomatic women. Timely detection not only improves survival but also extends the duration between diagnosis and death, regardless of whether immediate benefits are provided through screening.^27^ Most incidence-based cohort mortality studies, involving women who were invited for or attended screening, reported a reduction in breast cancer mortality.^5^ In a Swedish study, women participating in mammography screening had an estimated 41% reduction in the risk of dying from breast cancer within 10 years and an estimated 25% reduction in the rate of advanced breast cancer^10^ A Dutch study reported that a single invitation for breast cancer screening was effective in reducing breast cancer mortality.^13^ Additionally, a meta-analysis of 11 randomized trials reported a relative risk reduction in breast cancer mortality of 20% for women invited for screening compared with controls.^6^

Establishment of national screening programmes has sparked debate about their potential benefits and harms.^6^^,^^28^^,^^29^ Central to this discourse are considerations about mortality reduction, false-positive results, overtreatment, and accurate communication of risks and benefits.^30^^–^^34^ These differing views stem partly from debate over the validity of breast screening trials and the interpretation of observational data on breast cancer incidence and mortality.^27^^,^^31^^,^^35^^,^^36^ We did not evaluate the harms associated with breast screening programmes because of insufficient country-level data on false-positive results, overdiagnosis and overtreatment.

Differences in the availability of breast cancer screening programmes were evident between regions. Specifically, regions with a greater proportion of countries lacking regular screening programmes had a significant increase in breast cancer mortality. To further investigate this issue, we compared breast cancer mortality between countries with regular and irregular screening programmes within each region. However, some GBD regions, such as Australasia, high-income North America and southern Latin America, did not have any countries with irregular screening programmes, whereas other regions, such as central sub-Saharan Africa, lacked any country with regular screening programmes. Hence, we performed the analysis at the super-regional level, with regions nested within larger geographical subdivisions. Across the subdivisions, we observed varying effects of breast cancer screening programmes on breast cancer mortality. The differences in breast cancer mortality between countries with regular and irregular screening programmes across most of these larger regions were not significant. The analysis window of 6 years, including the COVID-19 pandemic period, may have been too short to capture the full impact of screening programmes effectively. However, in Latin America and the Caribbean, South-East Asia, East Asia and Oceania, and South Asia super-regions, countries with regular screening programmes showed significant reductions in age-standardized mortality rates compared with countries with irregular programmes.

The frequency of breast cancer screening in South-East Asia and Western Pacific regions varies significantly due to differences in health-care infrastructure, awareness and resources.^37^ Singapore, for example, has established organized mammographic screening programmes for women aged 50–69 years, with biennial screenings. Conversely, in Indonesia, Thailand and Viet Nam, organized screening is less prevalent. Thailand relies primarily on clinical breast examinations rather than mammography, underscoring resource and infrastructure limitations.^37^ Moreover, in women in South-East Asia, stage II tumours were most common at first diagnosis of breast cancer.^38^ A systematic review on breast cancer staging at diagnosis showed considerable heterogeneity among 22 countries of Latin America and the Caribbean, and reported that 40.76% (95% UI: 37.03–44.60) of women were diagnosed with stage III–IV breast cancer.^39^ In sub-Saharan Africa, 74.7% (18 087/24 213) of patients with breast cancer are diagnosed with stage III–IV disease.^40^ The stage at diagnosis is dependent on the efficiency of screening programmes, which partly explains the variations in mortality across countries.^39^

In the European region, where almost all countries have mammographic screening programmes, countries with higher screening coverage had a substantial reduction in age-standardized breast cancer mortality compared with countries with lower screening coverage. This reduction in mortality was particularly prominent in women aged 50–74 years, highlighting the association between mammographic screening and reduction in breast cancer mortality in the European region. 

Our study has limitations. First, an analysis over 6 years, including the COVID-19 pandemic period, may be too short to show the full benefit of screening programmes. This factor may explain why differences at the regional level were not fully apparent. Hence, studies with longer durations are needed. Nevertheless, evidence from randomized controlled trials indicates a reduction in mortality as a result of screening programmes within 4 years, with a progressively increasing effect up to 10 years.^41^ Second, we accessed qualitative data on breast cancer screening programmes at the country level from the WHO Global Health Observatory database, which has both mammographic screening and clinical breast examination data.^4^ Consequently, our analysis included countries with breast cancer screening programmes using either of these methods. Sufficient evidence exists showing that mammographic screening reduces breast cancer mortality in women aged 50–69 years and 70–74 years, but not for clinical breast examination.^5^ Nevertheless, clinical breast examination provides adequate evidence on stage shifts in breast tumours, indicating its potential for detecting tumour progression.

Third, data on breast cancer screening coverage across all countries and in subsequent calendar years are limited. Additionally, the database we used for breast cancer screening coverage did not provide information for all countries in the European region. Finally, no data were available on breast cancer subtypes; hence, we could not evaluate how subtype may have affected our findings. Screening alone is unlikely to account for differences in breast cancer mortality. We had no data on clinical and treatment-related factors and could not adjust for these factors. However, we adjusted for sociodemographic index, which may minimize potential confounding from sociodemographic status.

Breast cancer screening programmes are associated with reductions in breast cancer mortality, with regional differences in the distribution of these programmes and their impact on breast cancer mortality. Global efforts to reduce breast cancer mortality should include access to breast cancer screening programmes worldwide, particularly in resource-constrained settings.
